# Respiratory Syncytial Virus-Infected Mesenchymal Stem Cells Regulate Immunity via Interferon Beta and Indoleamine-2,3-Dioxygenase

**DOI:** 10.1371/journal.pone.0163709

**Published:** 2016-10-03

**Authors:** Michael B. Cheung, Viviana Sampayo-Escobar, Ryan Green, Martin L. Moore, Subhra Mohapatra, Shyam S. Mohapatra

**Affiliations:** 1 James A Haley Veterans Affairs Hospital, Tampa, Florida, United States of America; 2 Department of Molecular Medicine, University of South Florida Morsani College of Medicine, Tampa, Florida, United States of America; 3 University of South Florida College of Pharmacy, Tampa, Florida, United States of America; 4 Department of Pediatrics, Emory University School of Medicine, Atlanta, Georgia, United States of America; 5 Children’s Healthcare of Atlanta, Atlanta, Georgia, United States of America; Imperial College London, UNITED KINGDOM

## Abstract

Respiratory syncytial virus (RSV) has been reported to infect human mesenchymal stem cells (MSCs) but the consequences are poorly understood. MSCs are present in nearly every organ including the nasal mucosa and the lung and play a role in regulating immune responses and mediating tissue repair. We sought to determine whether RSV infection of MSCs enhances their immune regulatory functions and contributes to RSV-associated lung disease. RSV was shown to replicate in human MSCs by fluorescence microscopy, plaque assay, and expression of RSV transcripts. RSV-infected MSCs showed differentially altered expression of cytokines and chemokines such as IL-1β, IL6, IL-8 and SDF-1 compared to epithelial cells. Notably, RSV-infected MSCs exhibited significantly increased expression of IFN-β (~100-fold) and indoleamine-2,3-dioxygenase (IDO) (~70-fold) than in mock-infected MSCs. IDO was identified in cytosolic protein of infected cells by Western blots and enzymatic activity was detected by tryptophan catabolism assay. Treatment of PBMCs with culture supernatants from RSV-infected MSCs reduced their proliferation in a dose dependent manner. This effect on PBMC activation was reversed by treatment of MSCs with the IDO inhibitors 1-methyltryptophan and vitamin K_3_ during RSV infection, a result we confirmed by CRISPR/Cas9-mediated knockout of IDO in MSCs. Neutralizing IFN-β prevented IDO expression and activity. Treatment of MSCs with an endosomal TLR inhibitor, as well as a specific inhibitor of the TLR3/dsRNA complex, prevented IFN-β and IDO expression. Together, these results suggest that RSV infection of MSCs alters their immune regulatory function by upregulating IFN-β and IDO, affecting immune cell proliferation, which may account for the lack of protective RSV immunity and for chronicity of RSV-associated lung diseases such as asthma and COPD.

## Introduction

Respiratory syncytial virus (RSV) is the most common cause of respiratory tract infection in infants and young children as well as a frequent cause of pneumonitis and death in elderly and immunocompromised adults. According to the CDC RSV accounts for between 100,000 to 126,000 hospitalizations annually in children under one year old and each year, on average, 177,000 hospitalizations and 14,000 deaths are attributed to RSV infections in US adults over the age of 65 [1]. An enveloped single stranded RNA virus of the genus *Pneumovirus*, RSV has been associated with airway remodeling, immune system modulation and more severe airway hyper-reactivity in asthma [2–9]. Virtually all infants have been infected with RSV by the age of 2, however prior exposure does not produce protective immunity and recurrent infections even within the same RSV season are possible [10]. The molecular and immunologic basis for the lack of protective immunity after primary infections with RSV remains unclear.

The original formalin-inactivated vaccine developed in the 1960s lead to enhanced inflammatory disease and death of infants [11, 12] and despite the decades of study since there is still no FDA approved vaccine for RSV [13, 14]. A better understanding of the mechanisms of RSV immune evasion may facilitate the development of a safe and effective vaccine. Prophylaxis with Palivizumab, a monoclonal antibody against the viral fusion protein, has been used in high-risk infants since the late 1990s and is associated with reduced hospitalization due to lower respiratory tract infections, however no reduction in infant mortality or infection rate has been shown and the utility of the drug is limited due to exorbitant cost, $21.10 per mg at a recommended dose of 15 mg/kg, or approximately $1,435 per dose for a 10 lb infant excluding associated healthcare costs such as office visit fees for a pediatric pulmonary specialist [15, 16]. These injections only provide passive immunity and are required monthly throughout the RSV season, typically 5 months in most regions of the US not including Florida [8], and are often required for the first two years of life.

Until recently RSV infections were assumed to be confined to the apical airway epithelial cells, however, *in vitro* and animal models have shown that RSV can infect beyond the apical layer of airway epithelial cells through physical damage to the epithelium as well as epithelial cell denuding and sloughing due to the infection [17, 18]. Further, recent reports of extrapulmonary manifestations of RSV in humans have revealed that the virus is capable of infecting various immune cells of blood and bone marrow. Specifically, replicating RSV and RSV transcripts have been identified in blood neutrophils, dendritic cells, as well as human bone mesenchymal stem cells, also known as multipotent mesenchymal stromal cells (MSCs) [19–24]. Infectivity of MSCs is of particular interest since they can be found throughout the body in many tissues and are involved in immune regulation and tissue regeneration [25]. MSCs are known to mobilize to sites of injury for tissue repair [26–28] and have been identified as a major cell type responsible for regulating immune responses via a number of factors including indoleamine-2,3-dioxygenase. MSCs are found in nearly every vascularized tissue of the body including areas known to come into contact with RSV such as the lung and upper respiratory tract [29–32]. Also, the detection of RSV in marrow-derived MSCs suggests that the bone marrow may provide RSV with an immune-privileged site to evade or influence the host response and a staging area for potential subsequent RSV infections and chronic inflammatory disorders.

The increased prevalence of RSV infection in transplant patients and growing interest in utilizing MSC infusions for therapeutic purposes, including solid organ transplantation, nerve cell and tissue regeneration, as well as in control of autoimmune disorders [33–36], warrants a better understanding of the role of RSV infected MSCs in inflammation and immunity. Our initial studies revealed that RSV readily infects human MSCs; 1 MOI of virus led to nearly complete infection (greater than 90%) of MSC cultures compared to approximately only 40% of normal human bronchial epithelial cell cultures. This led us to hypothesize that RSV infection of resident MSCs as well as those mobilized by inflammation in the lung and respiratory tract [37, 38] may play a role in increasing the spread of RSV in the lung while limiting the robustness of the innate and adaptive immune responses. To test this, we undertook a comprehensive analysis of virus replication, gene transcription and protein expression in MSCs and examined the RSV-induced expression of cytokines, viral response factors such as type I and II interferon, and immune regulatory factors, such as iNOS and IDO. We then examined whether RSV-infected MSCs affect the proliferative capability of lymphocytes. The results show that RSV infected MSCs exhibit increased expression of immune regulatory factors and may play a role in mediating viral pathogenesis via immune tolerance.

## Materials and Methods

### Cell lines

**Fig 1:**
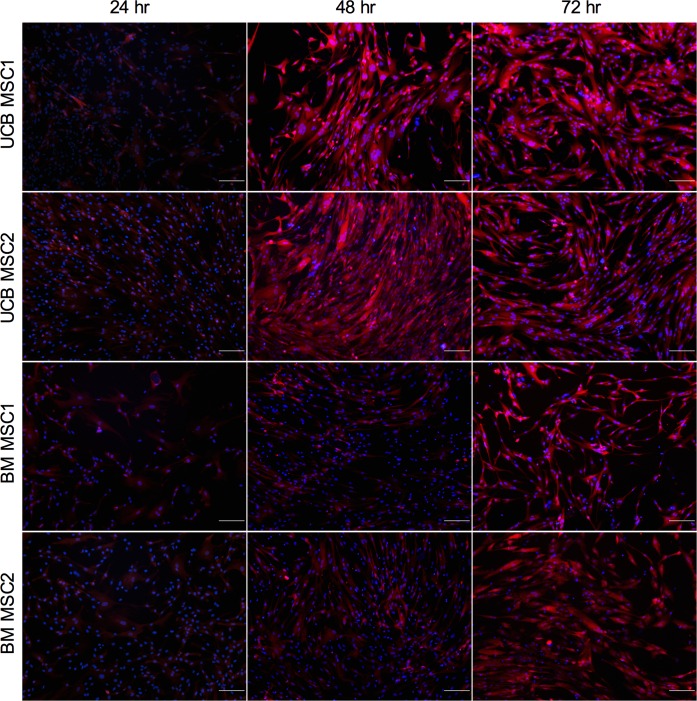
RSV infection of human MSCs. Two lots of UCB MSCs as well as two lots of BM MSCs were shown to become infected with RSV expressing mKate2. Live cell images were captured by fluorescent microscopy at 24, 48, and 72 hours post-infection using a fixed exposure time of 1/15 seconds for both blue and red filters with auto black balance, merged. 100X total magnification, scale bar 200μm, mKate2 (red), NucBlue live cell nuclear stain (blue). A representative picture from each experiment is shown.

Two lots of human umbilical cord blood MSCs (UCB MSC, Vitro Biopharma, Golden, CO, USA) and two separate lines of human bone marrow-derived MSCs (BM MSC, Institute for Regenerative Medicine at Scott & White Texas A&M Health Science Center College of Medicine, Temple, TX, USA) were tested. MSCs were cultured in complete MSC media: alpha-MEM with 1X Glutamax (Life Technologies, Carlsbad, CA, USA), 16.5% fetal bovine serum (FBS), 100 units/ml penicillin and 100 μg/ml streptomycin (Life Technologies). MSCs were passaged no more than 10 times before use in any experiment. For consistency, excepting Fig 1, all results shown were obtained from one of the UCB MSC lines. All four MSC lines showed similar results in triplicate experiments. Normal human bronchial epithelial (NHBE) cells (Lonza, Allendale, NJ, USA) were grown in BEGM (Lonza) and used prior to passage 5 in all experiments.

### Respiratory syncytial virus strain and PFU assay

The strain of RSV used in all except the lentiviral experiments was a recombinant A2 strain expressing a red fluorescent marker, mKate2, as well as the F protein from the clinical strain Line 19 (rA2-KL19F) [39, 40]. For visualization of viral infection in lentiviral-transduced cells a recombinant green fluorescent protein-expressing RSV was used (generously provided by Dr. Mark Peeples, Nationwide Children’s Hospital, Columbus, OH) [41]. Viral stocks were prepared by propagating the virus in HEp-2 cells (ATCC, Manassas, VA, USA) and purification by ultracentrifugation in a 30% glycerol, 0.1M MgSO_4_ and 50mM HEPES solution for 3 hours at 24,000×g. Viral pellet was washed and resuspended in precooled buffer: 0.1M MgSO_4_, 150mM NaCl and 50mM HEPES (Sigma-Aldrich, St. Louis, MO, USA). This buffer was used as mock infection media in all experiments. Viral stocks were stored in small aliquots at -80C and never thawed more than once.

The number of infective units was determined by PFU assays performed in the HEp-2 cell line. Cells were grown to confluence in 24-well plates, washed and incubated for 90 minutes with serial dilutions of purified virus stock in Opti-MEM with 2% FBS. Cells were washed and covered in DMEM media containing 5% FBS and 0.8% methylcellulose. After 5 days cells were fixed in 4% PFA and plaques were visualized and counted with a fluorescent microscope. Results were the average of 4 wells.

### RSV infection of human MSCs

To demonstrate the infection of MSCs with RSV: passage 3 cells were plated at 3 x 10^5^ cells per well in a 6 well plate with a sterilized 25mm round coverslip in the well. After 24 hours cells were infected with 10^6^ PFU of RSV (1 MOI) in Opti-MEM with 2% FBS or mock infection media for 90 minutes at 37C. Infection media was then aspirated and cells were washed once before incubation with complete MSC media for 48 or 72 hours. Media was aspirated, cells were washed with PBS and the coverslip was removed and fixed in cold acetone before staining for RSV (#5022 Mouse anti-RSV+FITC monoclonal kit; Millipore, Billerica, MA, USA). Coverslip was mounted on a slide upside down with mounting media containing 4,6-diamidino-2-phenylindole (DAPI; Vector Laboratories, Burlingame, CA, USA).

### Quantitative PCR assay

To measure RSV virus replication in human MSCs cells were infected with 1 MOI RSV (or mock) as previously described. Supernatants as well as RNA were collected 72 hours post-infection. Trizol method was used to isolate RNA (Invitrogen, Carlsbad, CA, USA) that was reverse transcribed to DNA (Maxima first stand kit; Thermo Fisher Scientific, Waltham, MA, USA) for use in qPCR. RSV transcripts were measured using SYBR green (Life Technologies) and normalized to GAPDH expression (Table 1; Integrated DNA Technologies, Coralville, IA, USA).

**Table 1:** Real-time RT-PCR primers.

Gene	Ref Seq	Primer	Sequence (5'-3')	Amplicon	Exon
GAPDH	NM 002046.3	IDT 103685527	CCACATCGCTCAGACACCAT	87	2 to 3
		IDT 103685147	AAAAGCAGCCCTGGTGACC		
RSVN	M74568.1	IDT 103685155	CATCTAGCAAATACACCATCCA	70	N/A
		IDT 103686014	TTCTGCACATCATAATTAGGAGTATCAA		
IL1B	NM 000576	IDT Hs.PT.51.20299051	AGGAGCACTTCATCTGTTTAGG	140	1 to 4
			GCCAATCTTCATTGCTCAAGTG		
IL-6	NM 000600.3	IDT 103685150	GATGGCTGAAAAAGATGGATGC	184	3 to 4
		IDT 103705045	CTGCAGGAACTGGATCAGGACT		
IL8	NM 000584	IDT Hs.PT.51.755000.g	ACACAGAGCTGCAGAAATCAG	144	1 to 1
			TTTCAGAGACAGCAGAGCAC		
CXCL12	NM 000609	IDT Hs.PT.51.2268843	CATCTGTAGCTCAGGCTGAC	89	1 to 2
			ATGAACGCCAAGGTCGTG		
IFNA2	NM 000605	IDT Hs.PT.53a.24294810.g	TTGACTTGCAGCTGAGCA	94	1 to 1
			CCCATTTCAACCAGTCTAGCA		
IFNB	NM 002176	IDT Hs.PT.53a.28162302.g	GTCAAAGTTCATCCTGTCCTTG	136	1 to 1
			CTCCACTACAGCTCTTTCCAT		
IFNG	NM 000619	IDT Hs.PT.53a.2672945	CGACAGTTCAGCCATCACTT	113	3 to 4
			GCAACAAAAAGAAACGAGATGAC		
IL1RN	NM 000577	IDT.Hs.PT.51.4381999	TTGTCCTGCTTTCTGTTCTCG	89	7 to 8
			CTGTCCTGTGTCAAGTCTGG		
IDO1	NM 002164	IDT Hs.PT.51.3356559	ACGTCCATGTTCTCATAAGTCAG	125	4 to 6
			GTTCCTTACTGCCAACTCTCC		
NOS2	NM 000625	IDT Hs.PT.51.22593297	GAGCTCAGATGTTCTTCACTGT	99	1 to 2
			AAGTTCTCAAGGCACAGGTC		
mGAPDH	NM 008084	IDT Mm.PT.39a.1	GTGGAGTCATACTGGAACATGTAG	150	2 to 3
			AATGGTGAAGGTCGGTGTG		
mIFNB1	NM 010510.1	IDT Mm.PT.56a.30132453.g	GGCATCAACTGACAGGTCTT	119	1 to 1
			ACTCATGAAGTACAACAGCTACG		
mIDO1	NM 008324	IDT Mm.PT.58.42364388	AAGCTGCCCGTTCTCAATC	127	1 to 2
			AGACCACCACATAGATGAAGATG		
mNOS2	NM 001313922	IDT 134627193	CCAAGCCCTCACCTACTTCC	127	15 to 16
		IDT 134627194	CTCTGAGGGCTGACACAAGG		

For cytokine expression RNA from RSV (or mock)-infected MSCs was collected 72 hours post-infection as previously described. Transcripts for the cytokines IL-6, IL-1β, IL-8, and stromal cell-derived factor 1 (SDF-1) as well as the viral response genes interferon (IFN)-α, IFN-β, and IFN-γ were quantified by RTqPCR and normalized to GAPDH (Table 1; Integrated DNA Technologies).

To assay expression of immune regulatory factors produced by MSCs mRNA transcript levels were assessed including indoleamine 2,3-dioxygenase (IDO), nitric oxide synthase (iNOS), and IL-1 receptor antagonist (IL-1ra) by RTqPCR at 72 hours post infection (Table 1; Integrated DNA Technologies).

### PFU assay

Clarified supernatants were used for PFU assays. HEp-2 cells were grown to confluence before infection with serial dilutions of supernatants from infected or mock-infected MSCs in Opti-MEM with 2% FBS. After a 90-minute incubation at room temperature with rocking the supernatants were removed and cells were cultured in DMEM containing 5% FBS and 0.8% methylcellulose for five days at 37°C in 5% CO_2_. Cells were fixed in 4% paraformaldehyde in PBS and plaques were counted using red fluorescence.

### Protein isolation

Cell lysates from infected or mock infected MSCs were collected following the method of Rudolph *et al* [42]. Briefly, cells were washed once with cold PBS, detached by scraping, and pelleted by centrifugation before adding freeze-thaw lysis buffer containing 600 mM KCl, 20 mM Tris-Cl (pH 7.8), 20% glycerol, and 1x each of protease and phosphatase inhibitor (EMD Chemicals Inc., San Diego, CA, USA). Samples were submerged in liquid nitrogen for 5 minutes and thawed on ice for 5 minutes three times, then centrifuged at 13,200 x g for 5 minutes to pellet debris. BCA protein analysis (Pierce Biotechnology, Rockford, IL, USA) was performed on the supernatant to measure the protein concentration before use in Western blots and IDO ELISA or enzyme activity assays.

### Western blotting

Cell lysates were separated using SDS-polyacrylamide gel electrophoresis and transferred onto nitrocellulose membranes. Membranes were blocked in Tris-buffered saline (TBS; 0.05 M Tris-HCl and 0.2 M NaCl, pH 7.4) containing 0.1% (v/v) Tween-20 (TBST) and 5% non-fat milk (w/v) for 1 hr at room temperature then probed with the appropriate primary antibody; either mouse anti-human IDO (RayBiotech, Norcross, GA, USA) or mouse anti-human beta-actin (Sigma-Aldrich, St Louis, MO, USA) diluted in TBST containing 5% non-fat milk. Probing was performed overnight at 4°C. After rinsing the membranes were incubated with HRP-conjugated anti-mouse IgG for 1 hr at room temperature, and then rinsed again. Band size was determined by comparison with a biotinylated protein ladder (Cell Signaling Technology Inc., Danvers, MA) and the density of each band was determined using ImageJ image analysis software [43] using a method detailed by Miller [44]. Results are reported as the relative abundance: a ratio of the density of the IDO band to the beta-actin band.

### ELISA

Conditioned media from MSCs infected with 1 MOI of RSV as previously described was clarified by centrifugation and the concentration of the immune regulator prostaglandin E_2_ (PGE_2_) was measured by ELISA (R&D Systems, Minneapolis, MN, USA). Cell lysates were obtained by freeze-thaw as described above for use in the ELISA kit for IDO (Cloud-Clone Corp., Houston, TX, USA).

### IDO enzymatic activity assay

To assess the activity of IDO we performed a tryptophan catabolism assay. Briefly, freeze-thaw lysates from RSV-infected MSCs were treated with buffer containing 100mM PBS, 40mM ascorbate, 20uM methylene blue, 200ug/ml catalase, and 800uM L-tryptophan (pH 6.5). After a 30 minute incubation at 37C the reaction was stopped with 30% trichloroacetic acid and heated to 52C for 30 minutes. Precipitate was removed and kynurenine in the supernatant was detected with Ehrlich’s reagent (0.8% p-dimethylaminobenzaldehyde in acetic acid) by reading absorbance at 490nm. Results are expressed as units per μg lysate where 1 unit of IDO activity is defined as the amount of enzyme producing 1nmol per hour of kynurenine.

### Isolation of PBMCs and cell proliferation assay

To examine the effect of RSV infection on MSC regulation of lymphocyte responses peripheral blood mononuclear cells (PBMCs) were isolated from fresh buffy coats (Florida Blood Services, St. Petersburg, FL, USA) and labeled with carboxyfluorescein succinimidyl ester (CellTrace CFSE, Life Technologies). Stained cells were then stimulated with the T cell mitogen phytohemagglutinin (PHA) in the presence of conditioned media (CM) from MSCs, infected 48hrs prior. Proliferation of lymphocytes was assessed by flow cytometry 5 days later using an LSRII flow cytometer (BD Biosciences, San Jose, CA, USA). Results were analyzed using FlowJo software (Treestar, Ashland, OR, USA).

### Inhibition of IDO in RSV-infected MSCs

In order to reverse the effects of IDO on PBMC proliferation MSCs were infected with RSV as previously described. After removing infection media MSCs were incubated in complete MSC media containing the IDO-inhibitor 1-methyltryptophan (1-MT, Santa Cruz Biotechnology) at 1mM. After 48 hours supernatants clarified by centrifugation were used in the PBMC proliferation assay as previously described. An alternative IDO inhibitor, vitamin K_3_ (menadione, Sigma-Aldrich, St Louis, MO, USA) [45] was used at 10μM to confirm our results. Additionally, to interrogate the pathways leading to IDO expression we treated RSV infected MSCs with either the endosomal pathway inhibitor chloroquine (10μg per mL, Sigma-Aldrich), the RIG-I pathway inhibitor BX795 (1μM, Invivogen, San Diego, CA, USA) or a neutralizing antibody to IFN-β (IFNb/A1, 10μg per mL, BioLegend, San Diego, CA, USA). To examine endosomal TLRs specifically we treated human MSCs with inhibitory oligodextronucleotide (ODN) 2088, an inhibitor of TLR7/8/9 [46–48] (50 μg per mL, Innaxon, Tewkesbury, UK) [49], or TLR3/dsRNA complex inhibitor (50 μM, Calbiochem, San Diego, CA, USA) [50]. With each inhibitor or vehicle, MSCs were pretreated overnight before infection with RSV. Following infection MSCs were incubated in fresh media containing the inhibitors or vehicles. After 24 hours additional inhibitor was added and at 48 hours samples were collected for RNA.

### Knockdown of IDO in RSV-infected MSCs

Human MSCs were transduced with lentiviral particles containing the pCRISPR-LvSG03 expression plasmid for mCherry, CRISPR associated protein 9 (Cas9), and one of three human IDO-specific single guide (sg)RNAs or a scrambled control (LPPHCP255475L03-3-a-100 and LPPCCPCTR01L03-100, GeneCopia Inc., Rockville, MD, USA). MSCs were seeded into 6 well plates at a density of 10,000 cells per cm^2^. Cells were allowed to attach overnight and were transduced with one of 4 lentiviral constructs the following day in the presence of 10 μg per mL polybrene for 12 hours, per manufacturer’s instructions. The cells were incubated for 48 hours prior to infection with rgRSV. RSV infection and lentiviral transduction were visualized by fluorescent microscopy for GFP (green) and mCherry (red) respectively.

### RSV infection of IDO-deficient mice

Wild type C57Bl/6 mice (WT) as well as mice deficient in IDO (IDO-KO) were purchased from Jackson Laboratories (B6.129-Ido1^tm1Alm^/J, # 005867) and infected with RSV when they were 6–8 weeks old by intranasal administration of 1 million PFU. Five days post infection the mice were euthanized and lungs were collected and processed for histology, plaque forming unit assay, and RNA quantification as previously described [51]. The right bronchus was clamped shut while the left lung was perfused with 4% paraformaldehyde (PFA) in PBS via the trachea, then removed to a vial containing 4% PFA for histology. The right lobes were removed, minced, portioned into two 1.5 mL tubes, and snap-frozen on dry ice for RNA and plaque forming unit assays. All animal procedures were reviewed and approved by the University of South Florida’s Institutional Animal Care and Use Committee.

### RSV infection of mouse MSCs

MSCs were isolated from the bones of wild type C57Bl/6 mice using the method of Nardi, *et al*. [52, 53]. Hind leg bones were excised, cleaned, and flushed with PBS containing 1% FBS. Contaminating red blood cells were lysed and adherent cells were grown for 3 passages. Cells were characterized by staining for the MSC markers CD105, CD29, and Sca-1 as well as the CD45 lineage surface markers (Mouse Mesenchymal Stem Cell Multi-Color Flow Kit, R&D Systems) as well as their capacity to differentiate, as described previously [54]. Mouse MSCs were infected as previously described, with 1 MOI RSV. RNA was collected 72 hours post-infection and the transcripts for mouse IFN-β, IDO, and iNOS were measured (Table 1, Integrated DNA Technologies).

### Statistical analysis

All experiments were repeated at least twice depending upon the type of experiments. Statistical significance for each experiment was determined by Student’s t test or one-way analysis of variance (ANOVA) as appropriate with an alpha of 5%. Calculations were performed and graphs produced using Prism 6.0 software for Mac OS (Graphpad Software, San Diego, CA, USA). Graphs of results show the mean and error bars depict the standard deviation.

## Results

### RSV infects and replicates in human MSCs

**Fig 2:**
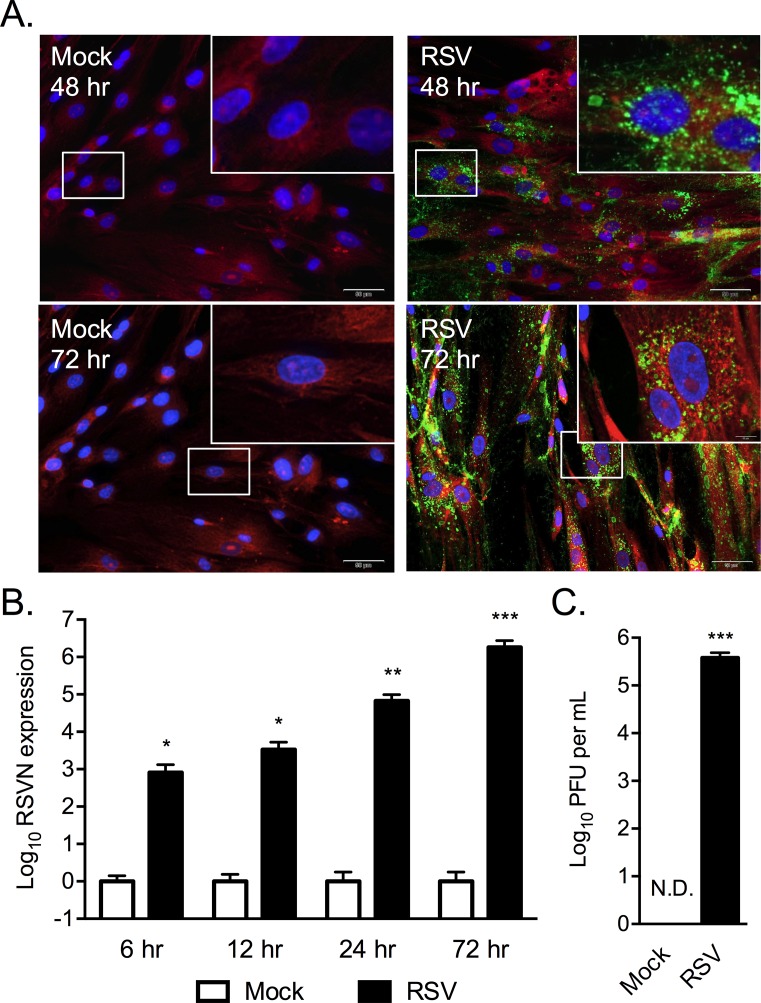
RSV infects and replicates in human MSCs. Human MSCs grown on coverslips, infected with 1 MOI of RSV, and immunostained at 48 or 72 hours post-infection with a monoclonal antibody to RSV tagged with FITC (green), Evan’s blue dye (red) and DAPI (blue). Total magnification of images is 200X (inset 1000X) and scale bar is 50μm (A). RSVN transcripts detected in MSCs at 6, 12, 24 and 72 hours post infection normalized to mock (B). RSV titers (PFU/ml) isolated from the culture medium of infected and mock-infected MSCs is shown (C). Results representative of at least duplicate experiments. * p < 0.05, ** p < 0.01, *** p < 0.001.

Live cell imaging of the infected cells for m-Kate2 expression at 24, 48 and 72 hours post-infection demonstrated RSV was capable of infecting four different MSC lots including two UCB MSC lines and two BM MSC lines (Fig 1). FITC-labeled anti-RSV antibody detected the viral products in MSCs at 48 and 72 hours post-infection (Fig 2A). The antibody used was a mixture of 4 monoclonal antibodies derived from mice against the nucleocapsid protein (N), glycoprotein (G), and fusion protein (F, 2 antibodies), all tagged with FITC. RTqPCR detected RSV specific transcripts for the nucleocapsid gene at 5.8 million-fold in the RSV infected MSCs after 72 hours compared with uninfected A549 control (P = 0.0498 by one sample t test) while levels in mock infected MSCs were not significantly different (Fig 2B). Culture supernatants isolated from RSV infected MSCs 72 hours after infection and used in PFU assays with HEp-2 target cells contained a mean of 3.8 X 10^5^ PFU/ml in the infected cultures compared to mock, where no plaques were detectable (p < 0.0001 by Wilcoxon signed-rank test; Fig 2C).

### RSV infection induces differential cytokine expression in MSCs and epithelial cells

**Fig 3:**
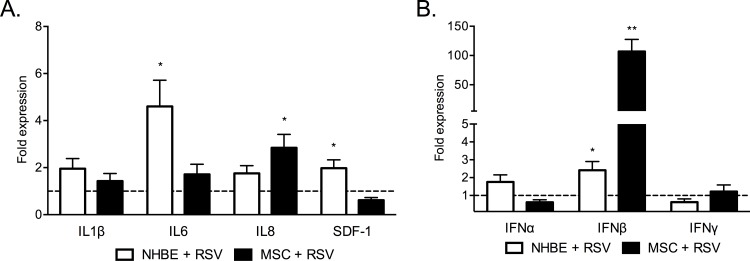
RSV infection increases IFN-β transcription in human MSCs. Cytokine transcription in RSV-infected NHBE cells and MSCs (n = 6) after 72 hours post infection by RTqPCR reported as fold expression compared to mock-infected NHBE and MSCs. (A). Interferon transcript expression in RSV-infected NHBE and MSCs compared to mock-infected cells (n = 6) (B). Results of a representative experiment. * p < 0.05, ** p < 0.01.

RNA from RSV infected NHBE cells and MSCs was examined for the expression of pro-inflammatory cytokines, IL-1b, IL-6, IL-8, SDF-1, and type I and type II interferons. At 72 hours post-infection RSV-infected NHBE cells had increased expression of IL-6 (4.6-fold, p = 0.0220) and SDF-1 (2-fold, p = 0.0457) transcripts compared to mock infected cells (Fig 3A). Transcripts for these cytokines were unchanged in RSV infected MSCs, however a 2.85-fold increase in IL-8 mRNA (p = 0.0291) was seen. An increase in IFN-β transcripts was observed for both RSV infected NHBE cells (2.409-fold, p = 0.0334) and MSCs (106.9-fold, p = 0.0036) compared to mock (Fig 3B). However, no changes in either IFN-α or IFN-γ expression were seen for either cell type.

### RSV infection increases expression of indoleamine-2,3-dioxygenase by MSCs and epithelial cells

**Fig 4:**
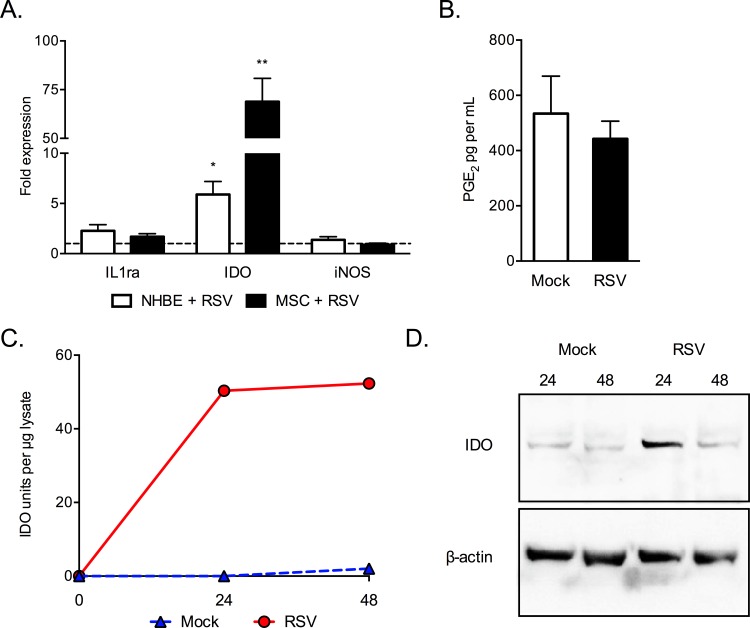
RSV infection of MSCs modulates expression of the immune regulatory factor IDO. Expression of IL-1Ra, IDO and iNOS were examined in RSV- or mock-infected NHBE cells and MSCs after 72 hours post infection by RTqPCR, reported as fold expression compared to mock (A). PGE2 expression was assessed by ELISA (B). (C-D) Tryptophan catabolism was measured in MSC lysates at 0, 24 and 48hrs after infection with RSV or mock. IDO activity (C) and expression by Western blot (D) is shown. * p < 0.05, ** p < 0.01.

RNA from RSV infected NHBE cells and MSCs was examined for the expression of the immune regulatory factors IL-1 receptor antagonist (IL-1ra), inducible nitric oxide synthase (iNOS) and IDO (Fig 4A). RSV infection increased IDO transcripts 68.9-fold compared to mock infected (p = 0.0023) in MSCs. A significant but less dramatic 5.9-fold increase was also observed for in RSV infected NHBE cells (p = 0.0120). No changes in IL-1ra or iNOS were seen. As another potential immune regulatory pathway, prostaglandin E_2_ (PGE_2_) level was measured by ELISA (Fig 4B). RSV-infection was not associated with any change in PGE_2_ levels in MSC culture supernatant.

IDO activity was detected in RSV-infected MSCs as 50.35 and 52.31 units per μg protein at 24 and 48 hours respectively, while it was undetectable at 24 hours and just 2.03 units per μg protein at 48 hours in mock-infected MSCs (Fig 4C). IDO expression in RSV-infected MSCs was confirmed by Western blot (Fig 4D). Analysis showed that IDO protein was detected 6-fold and 2-fold higher in RSV-infected MSCs compared with mock at 24 and 48 hours post infection, respectively. Cell lysates were also examined for IDO enzymatic activity by tryptophan catabolism assay.

### RSV induces IDO-mediated inhibition of PBMC expansion by MSCs

**Fig 5:**
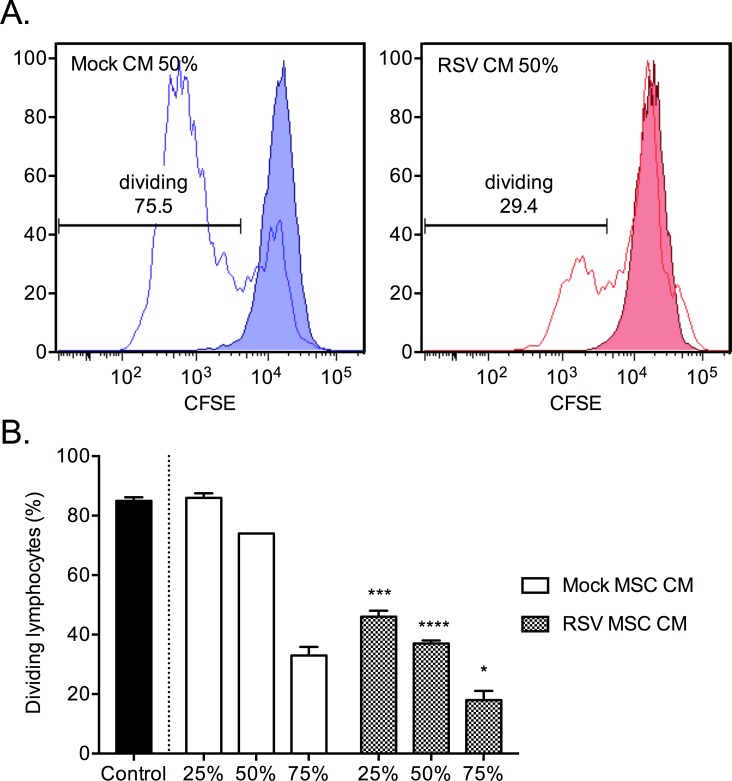
Conditioned media from RSV-infected MSCs reduces PBMC proliferation. Freshly isolated PBMCs stained with CFSE, treated with either RSV or mock conditioned media, and grown in triplicates with (line) or without PHA (shaded) for 5 days. CFSE fluorescence was measured by flow cytometry as an indication of proliferation (A). Dose-dependent reduction in proliferation of PBMCs treated with conditioned media from RSV-infected MSC (B). Result of a representative experiment is shown. * p < 0.05, *** p < 0.001, **** p < 0.0001.

To examine the immunological consequences of RSV-infected MSCs we isolated fresh human PBMCs and treated them with varying amounts of CM from MSCs. We observed a dose-dependent decrease in mitogen-stimulated proliferation of PBMCs treated with the CM from either RSV- or mock-infected MSCs (Fig 5). Additionally, the cells treated with CM from MSCs infected with RSV had significantly lower proliferation than those treated with mock at all concentrations tested (Fig 5B). PBMCs cultured with 25% CM from RSV-MSCs showed 40% less proliferation than those cultured with CM from mock-MSCs (46% vs. 86%, p = 0001). Similarly, PBMCs cultured with 50% or 75% CM showed a reduction in PHA-stimulated proliferation, 37% less (37% vs. 74%, P < 0.0001) and 15% less (18% vs. 33%, P = 0.0234), respectively. These results showed that RSV infection increased the regulatory effect of MSCs via factors in the CM. Since the lowest p-value was seen between the groups treated with 50% MSC CM we chose to use this concentration for future experiments.

**Fig 6:**
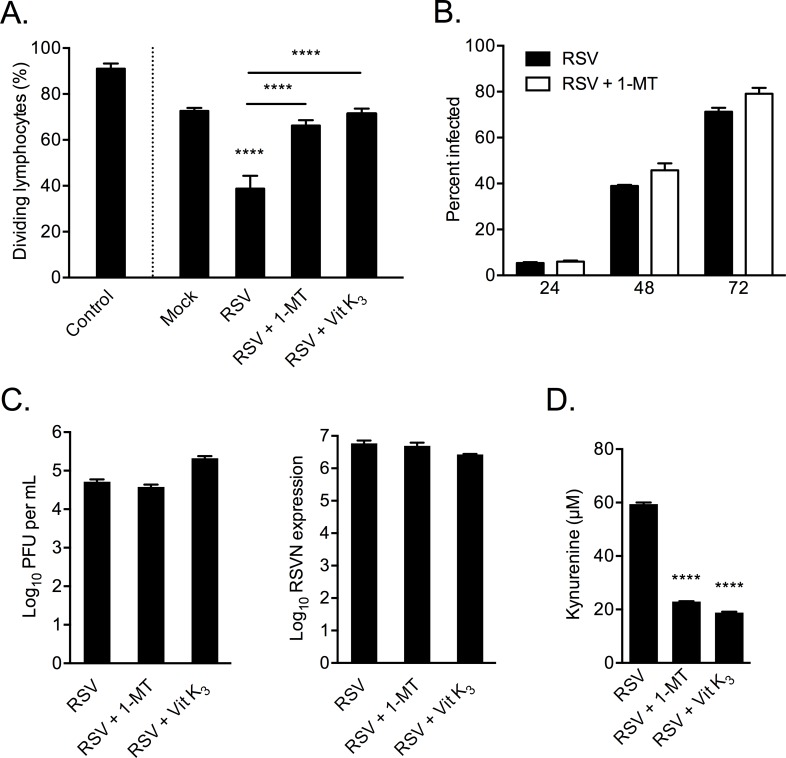
IDO antagonists abrogate proliferative inhibition by RSV-infected MSC conditioned media. Freshly isolated PBMCs were stained with CFSE, treated CM from MSCs, and cultured for 5 days with the mitogen PHA. Infected MSCs treated with 1-MT, vitamin K_3_ or mock were examined for their effects on PBMC proliferation by CFSE fluorescence in Flow cytometry (A). The reduction in MSC infection was examined by flow cytometry (B), and RSV infection was measured by PFU assay and RSVN expression (C). The effect of IDO inhibitors vs control (untreated) was examined in the supernatants of infected MSCs by kynurenine assay (D). Significance between groups determined by one-way ANOVA with Fisher’s LSD test. Results of a representative experiment of N = 2 are shown. **** p < 0.0001.

In order to investigate whether increased IDO production by RSV-infected MSCs was responsible for modulating PBMC expansion we used the IDO inhibitor 1-MT. MSCs were infected as previously, however in the treatment group infection media was replaced with MSC media containing 1mM 1-MT. The rest of the experiment was performed as described previously with PBMCs being treated with 50% MSC CM or MSC media as a control. PBMCs treated with CM from RSV-infected MSCs showed a significant reduction in mitogen-stimulated proliferation (38.77%) compared with mock (72.64%; P < 0.0001) while treatment of MSCs with 1-MT during RSV infection restored proliferative capacity of PBMCs (66.27%; versus mock CM P = 0.1657, versus RSV CM P < 0.0001) (groups compared by one-way ANOVA with Fisher’s LSD test, Fig 6A). Similar results were found when MSCs were treated with the alternative IDO inhibitor vitamin K_3_ (71.40%; versus mock CM P = 0.7771, versus RSV CM P < 0.0001). Flow cytometry, plaque assay and RTqPCR were used to show that the reversal was not due to inhibition of viral replication by the IDO inhibitor (Fig 6B–6D). These results demonstrate the potential immune regulatory role of IDO production by MSCs in RSV infection, an effect that was reversed in the presence of either of the IDO inhibitors.

### CRISPR-mediated knockout of IDO prevents anti-proliferative effects of RSV-infected MSCs

**Fig 7:**
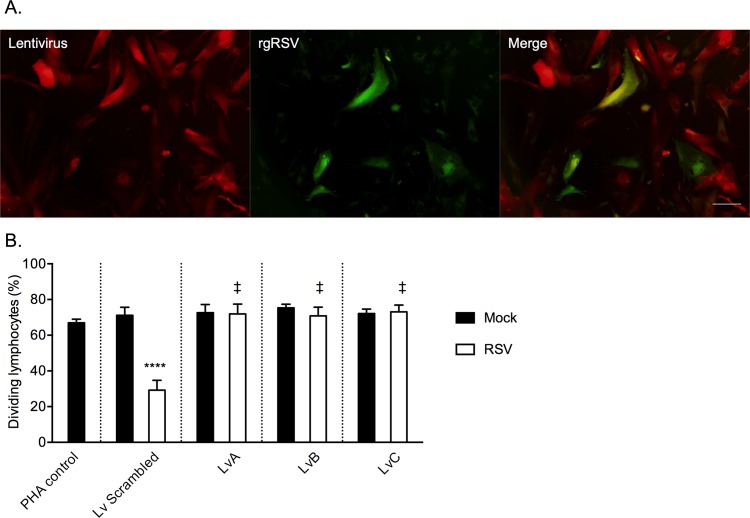
Knockout of IDO eliminates the immune regulatory effect of RSV-infected MSCs. Transfected MSCs were observed by fluorescent microscopy for lentiviral delivery of the plasmid (mCherry, red) as well as infection with rgRSV (GFP, green) (A). Proliferation of PBMCs treated with conditioned media from MSCs was detected by CFSE. Lv Scramled reresents media from MSCs transfected with Lv Scrambled construct; LvA, LvB, and LvC represent media from MSCs transfected with each of the three IDO-knockout constructs (B). N = 3, **** p < 0.0001 vs. PHA control, ‡ p < 0.0001 vs. Lv Scambled.

As an alternative method to the use of IDO inhibitors, which may have off target effects, we leveraged the CRISPR/Cas9 system to knockout the IDO gene from the human MSCs prior to infection with RSV. Three separate plasmids expressing different IDO-specific guide RNAs as well as a control plasmid expressing a non-targeted scrambled guide RNA were transfected individually into human MSCs. Expression of the plasmid was evident by fluorescent microscopy for mCherry (red) expression in cells (Fig 7A). Similarly, infection with rgRSV was observed by GFP (green) fluorescence (Fig 7A). Conditioned media from IDO-knockout and control MSCs was used in to treat PBMCs during a CFSE proliferation assay as previously described. PBMCs treated with conditioned media from infected MSCs that received the scrambled guide RNA (Lv Scrambled) showed a similar pattern of decreased proliferation due to RSV infection of the MSCs (Fig 7B). However, conditioned media from MSCs which lacked IDO expression (LvA, LvB, and LvC) showed no effect on PBMC proliferation regardless of RSV infection (Fig 7B). These results further demonstrate the role of IDO in immune regulation by RSV-infected MSCs.

### IFN-β neutralizing antibody reduces IDO expression

**Fig 8:**
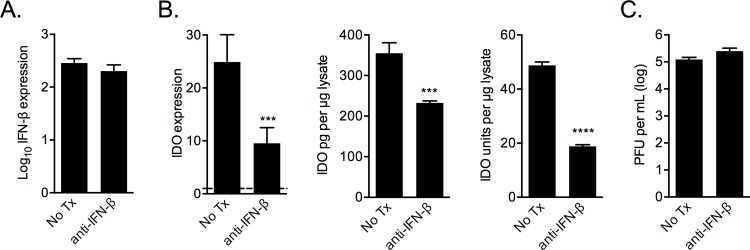
IFN-β antagonism reduces RSV-induced IDO expression and activity. RSV-infected MSCs were incubated with a anti-IFN-β neutralizing antibody and 24 h after were examined for IFN-β transcript levels (A), for IDO protein expression and enzymatic activity (B) and for plaque-forming units in the supernatant (C). Significance determined by one-way ANOVA with Fisher’s LSD test; n = 3. *** p < 0.001 **** p < 0.0001.

To examine the role autocrine IFN-β signaling may play in IDO expression RSV-infected MSCs were treated with a neutralizing antibody against IFN-β (anti-IFN-β). Treated MSCs had reduced IDO expression compared with untreated cells, which was not due to a reduction in IFN-β epression (Fig 8A). Further, treating the cells with either chloroquine or anti-IFN-β reduced IDO protein detected by ELISA as well as enzymatic activity in lysates from infected cells (Fig 8B). The decreased IDO was not due to an effect of the inhibitors on RSV infectivity or replication, measured by PFU production by infected cells (Fig 8C).

### Endosomal TLR but not RIG-I pathway inhibitors reduce IFN-β and IDO expression

**Fig 9:**
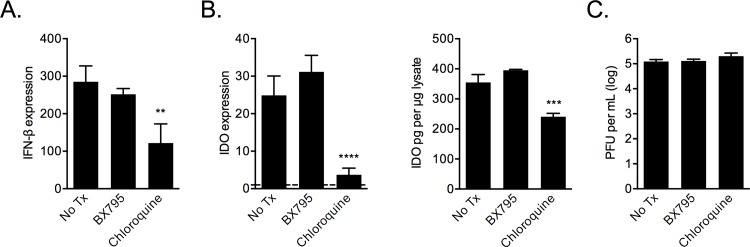
Endosomal and not RIG-I pathway inhibitors block IFN-β induction and IDO expression in RSV-infected MSCs. IFN-β levels were examined in infected MSCs treated with the endosomal pathway inhibitor chloroquine, vehicle control (No Tx), or with RIG-I pathway inhibitor BX795 (A). IDO protein expression was measured in cells treated with chloroquine versus BX795 (B), and cells were examined for RSV PFUs (C). Significance between groups determined by one-way ANOVA with Fisher’s LSD test; n = 3. ** p < 0.01, *** p < 0.001, **** p < 0.0001.

To examine the role of pathogen associated molecular pattern (PAMP) receptor signaling in IDO expression during RSV infection we treated infected MSCs with either the RIG-I signaling inhibitor BX795 or the endosomal TLR inhibitor chloroquine. Chloroquine treatment but not BX795 reduced IFN-β expression in RSV-infected human MSCs (Fig 9A). This reduction in IFN expression was associated with a reduction in IDO expression in RSV infected MSCs treated with chloroquine and was not seen in MSCs treated with BX975 (Fig 9B). Neither inhibitor was shown to effect the replication of the virus in MSCs (Fig 9C).

### Inhibition of TLR3 reduces IFN-β and IDO expression

**Fig 10:**
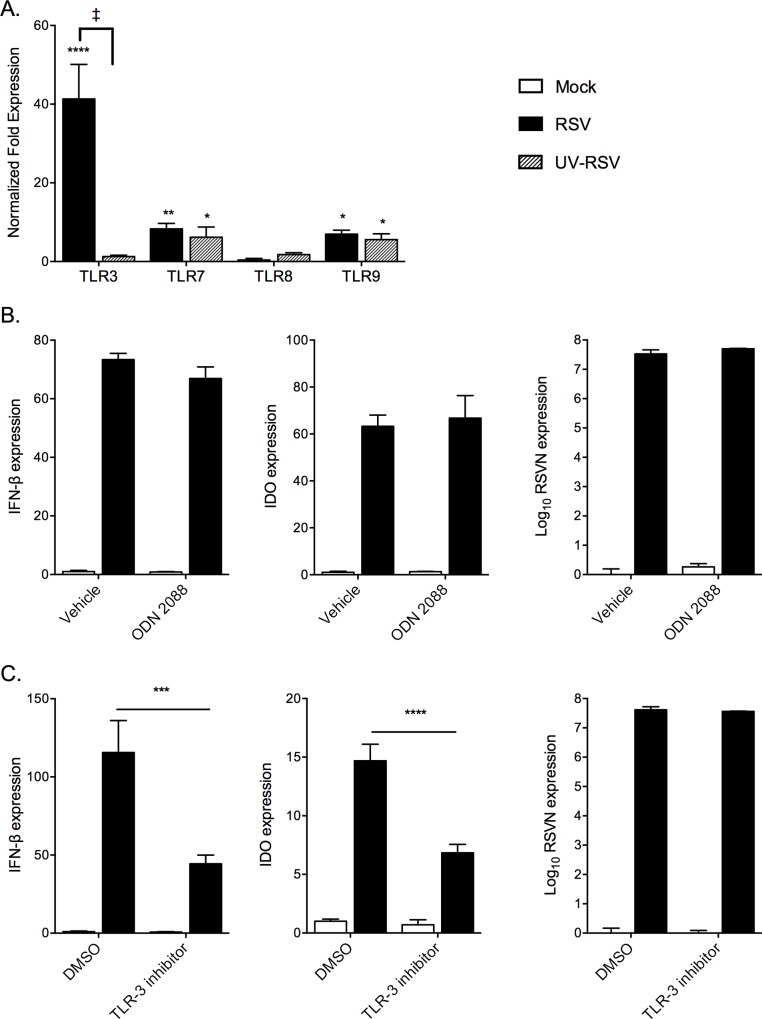
Antagonizing TLR3, but not TLR7/8/9, reduces RSV-stimulation of IFN-β and IDO. Expression of endosomal TLRs was accessed in MSCs infected with Mock, RSV, and UV-inactivated RSV (UV-RSV) (A). RSV vs Mock -induced IFN-β or IDO expression in infected MSCs was examined following treatment with TLR7/8/9 inhibitor ODN 2088 (B). Levels of IFN-β and IDO transcripts 48 hours post infection of MSCs was measured following treatment with the TLR3/dsRNA complex inhibitor versus DMSO (as control) (C). RSV infection was measured following treatment with TLR inhibitor vs mock-infected vehicle control by expression of RSV-N transcripts normalized to HPRT gene.; Statistical analysis by 2-way ANOVA; n = 3. * p < 0.05, ** p < 0.01, *** p < 0.001, **** p < 0.0001 vs. Mock, ‡ p <0.0001 vs. UV-RSV.

RSV stimulation of MSCs, either with live virus or UV-inactivated virus, induced endosomal TLR expression (Fig 10A); specifically TLR3, TLR7, and TLR9 were upregulated versus mock. However, only TLR3 was increased with live virus when compared to UV-inactivated control, suggesting its role in productive RSV infection. To examine the endosomal pathway or pathways responsible for the IFN-β response to RSV in MSCs we used two specific inhibitors; ODN 2088, an inhibitor of TLR7/8/9 activity [46–48], and TLR3/dsRNA Complex Inhibitor [50]. Treatment with ODN 2088 showed no effect on either IFN-β expression or IDO expression compared with vehicle treated cells (Fig 10B), suggesting that the TLR7, 8, and 9 pathways were not involved. However, when MSCs were treated with the TLR3/dsRNA complex inhibitor during RSV-infection they had reduced IFN-β and IDO (Fig 10C), suggesting a role for TLR3 in the observed response to RSV in MSCs. The effect of the inhibitor did not appear to be due to a change in viral replication, measured by RSV gene expression.

### RSV infection of IDO-deficient mice

**Fig 11:**
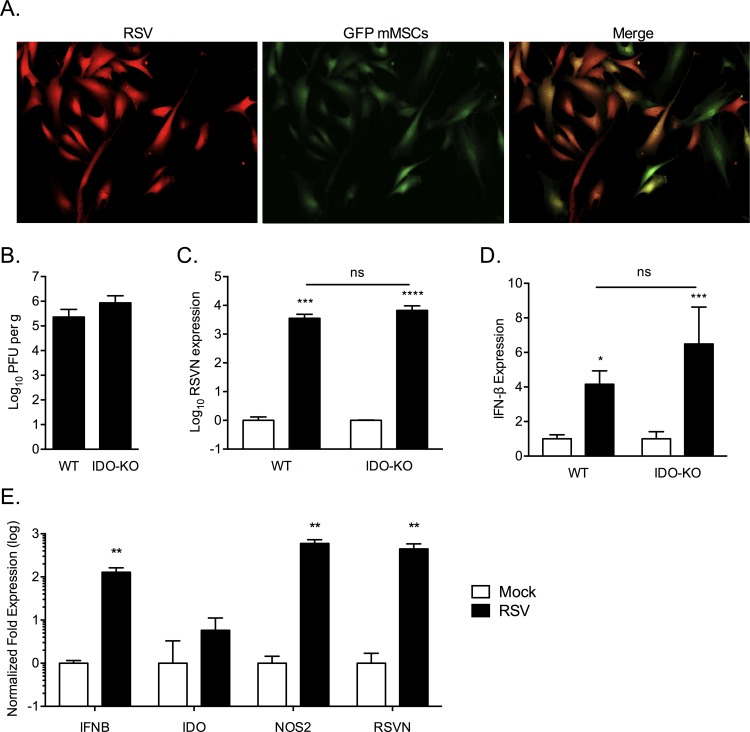
IDO-deficient mice are not protected from RSV infection. MSCs isolated from the leg bones of GFP- mice were infected by human RSV and visualized by fluorescent microscopy (A). RSV infection of wild type (WT) versus IDO knockout (IDO-KO) mice were compared by Plaque forming unit assay (B) and RSV-N gene expression (C). IFN-β expression was examined in the lungs of infected WT and IDO-KO animals (D). RSV- vs mock-infected mouse MSCs were examined for the expression of IFN-β,IDO, NOS2 and RSVN gene (normalized to HPRT) (E). Significance between groups determined by one-way ANOVA with Fisher’s LSD test. Results representative of duplicate experiments. * p < 0.05, *** p < 0.001, ns = non-significant.

After ensuring mouse MSCs could be infected *in vitro* with human RSV (Fig 11A) we infected WT and IDO-KO mice with 3 x 10^6^ PFU RSV intranasally. After 5 days infection we did not see any difference in viral load between WT and IDO-KO mice, as measured by PFU assay and RSVN mRNA in the lungs of infected mice (Fig 11B and 11C). There was also no difference in IFN-β expression (Fig 11D).

### RSV infection of mouse MSCs induces iNOS and not IDO

After the previous result we wanted to determine whether the mouse presented an appropriate model for examination of IDO expression by MSCs in response to RSV infection. Similar to human MSCs, wild type mouse bone marrow-derived MSCs infected with RSV exhibited a 128.2-fold increase of IFN-β transcripts (p = 0.0029), however IDO expression was unchanged (p = 0.23, Fig 11**E**). Interestingly, iNOS was highly transcribed following RSV infection at 594.1-fold the mock level (p = 0.0015). These results stand in contrast to what has been reported above in human MSCs (Fig 4) and suggest that RSV infection in mice, both *in vitro* and *in vivo*, may be an unsuitable model for examining the role of MSCs in RSV infections, at least as it pertains to IDO activity.

## Discussion

To date most RSV research has focused on the viral modulation of innate immune signaling within immortalized epithelial cell lines such as HEp-2 and A549 although recent reports of RSV infecting and replicating in cells of the immune system including blood and lung neutrophils [22, 55–57], macrophages [24, 58, 59], and dendritic cells [20, 21, 60] suggest that RSV is capable of infecting cells of non-epithelial origin. Though extrapulmonary RSV infection of MSCs in humans was reported [23, 61], little is known about its role in virus-mediated immune regulation and the lack of protective immunity during secondary RSV infection. Our study addresses the role of MSCs as a target cell for RSV and the consequences of this extra-epithelial infection and is the first to report an increase in the expression of the immune regulatory factors IFN-β and IDO.

Our findings demonstrate that RSV infects MSCs *in vitro* and infective virus progeny are produced by the MSCs. This observation is similar to the other reports [23, 61]. Infection of MSCs would require the virus to infect beyond the apical epithelial layer of the airway, a phenomenon that has been demonstrated in ovine and baboon animal models [17, 18]. *In vitro* air-liquid culturing of NHBE cells has allowed researchers to examine this further, demonstrating that RSV can gain access to and infect the basal progenitor cells beneath by epithelial denuding or physical damage to the apical cells [62, 63] as well as via transport by infected alveolar macrophages [64]. Similar mechanisms may permit RSV to gain access to airway stromal cells such as MSCs.

We sought to identify factors that could be important in mediating the effects of RSV in MSCs by examining differential expression of chemokines and cytokines involved in the inflammatory response. Unlike with RSV-infected NHBE cells, RSV-infected MSCs showed no change in the mRNA of the proinflammatory cytokines IL-6 or SDF-1 (CXCL12). However, there was a significant increase in the expression of the chemokine IL-8 (CXCL8), which is known for neutrophil recruitment and may be involved in mediating the inflammation and neutrophilia associated with severe RSV infections. Also, since neutrophils are susceptible to RSV [22] and are known to return to the bone marrow upon senescence [65, 66] they may contribute to the extrapulmonary RSV infection of bone MSCs observed by others [23, 61].

A major finding from our studies relates to modulation of interferon expression and signaling by RSV-infected MSCs. Our results showed that when infected with RSV both NHBE and MSCs displayed significant upregulation of IFN-β, but not in gamma or alpha interferon. Of note, MSCs showed a more than 100-fold increase in IFN-β transcript compared to mock as determined by RTqPCR. The functional relevance of this finding is unclear as RSV continues to replicate in MSCs and blocking IFN-β signaling with a neutralizing antibody did not increase virus replication. It is possible that the IFN-β mRNAs are sequestered in exosomes, or microvesicles, that are exported out of MSCs [44, 67, 68]. Alternatively, IFN-β-signaling simply may not be protective in the case of RSV infection of MSCs.

Another major finding from our studies is that although MSCs produce a number of immune regulatory factors including iNOS, IL-1Ra, and PGE_2_ [27, 28, 33, 69], none of these were altered in their expression during RSV infection. However, RSV-infected MSCs did show a significant increase in IDO transcripts and protein, as well as activity of the enzyme. Further, RSV-infected MSCs exerted a greater inhibition of mitogen-stimulated proliferation of human PBMCs through IDO activity. Antagonizing IDO activity with a competitive inhibitor such as 1-MT or vitamin K_3_ [45] reduced kynurenine levels in conditioned media of RSV infected MSCs and reversed the suppressive effect of MSC CM on lymphocyte proliferation, although these inhibitors did not affect RSV infective rate or proliferation in MSCs. These results suggest that the use of an IDO inhibitor during respiratory viral infection could result in a boosted immune response. This idea is consistent with a report which demonstrated that pretreatment of influenza-infected mice with 1-MT, increased inflammatory markers, such as IL-6, TNF-α and IFN-β [70], and Th1 cell numbers and boosted the secondary immune response [71].

Of special importance is our finding that there exists an autocrine signaling pathway in the regulation of IDO by IFN-β, both of which are significantly produced in RSV-infected MSCs and are also present in the proinflammatory environment of the lung during a respiratory viral infection. This notion is supported by our results that blocking IFN-β signaling with a neutralizing antibody resulted in reduced IDO expression and enzymatic activity in infected MSCs and agrees with reports in other immune cells showing an IFN-γ-independent pathway in the production of IDO involving IFN-β [72, 73]. It is possible this IFN-β-dependent pathway is also active in other cell types during RSV infection, such as dendritic cells and apical cells of the airway epithelium, which also play a role in regulation of the immune system and could be a target for blocking immune regulation by IDO during viral infection.

Regulation of immunity by MSCs has been linked to active TLR signaling [74–79] and products of RSV replication are known TLR ligands [80–82]. The results of our study showed chloroquine treatment of infected MSCs was sufficient to block IFN-β expression, which suggests that endosomal TLR recognition of viral products plays a key role in the MSC response to RSV infection. These results are similar to those reported for adenovirus and baculovirus, which induce an increased expression and TLR signaling in MSCs as well as increased IFN-β production [83]. Since RSV also stimulates immunity via the RNA helicase RIG-I through activation of the mitochondrial antiviral signaling complex (MAVS), TBK-1/IKKε, and IRF3 [9, 84], the role of this pathway in RSV infected MSCs was examined. Our results that the TBK1/IKKε inhibitor BX795 did not affect RSV-stimulated IFN-β expression suggest that the RIG-I pathway is not involved in this aspect of the MSC response to the virus. Further examination of the endosomal TLR pathway using ODN 2088, an antagonist of the endosomal TLR7/8/9 pathways, implied that while TLR7 and TLR9 were each upregulated versus mock infection with either UV-inactivated or live RSV neither played a role in the IFN-β or IDO expression we observed. However, MSCs treated with the TLR3/dsRNA complex inhibitor did show a reduction in IFN-β and IDO following RSV infection and TLR3 was the only TLR we observed to have significantly increased expression when comparing live infection to UV-inactivated virus. These results together suggest a role for endosomal TLR3 recognition of the virus in the expression of IDO, via IFN-β.

Our findings with MSCs differ from a recent report on IDO expression in recombinant green RSV-infected monocyte-derived dendritic cells (DCs) [42], which showed that the induction of IDO and IFN-γ were due to RIG-I pathway activity and not endosomal TLRs. Interestingly, in contrast to DCs RSV-infected MSCs showed an increase in IFN-β, not in IFN-γ. This result is similar to those previously reported in epithelial cells where RSV has been found to antagonize IFN-γ expression by inhibition of RIG-I pathway molecules via RSV nonstructural proteins [63, 85]. These discrepancies suggest that the cell types may differ in their activation of molecular pattern recognition receptors and subsequent signaling, perhaps due to their differential susceptibility to RSV. The convergence of both pathways in the induction of IDO is an interesting finding, regardless. Future work could focus on the different viral response pathways triggered by classical antigen presenting cells such as monocytes and DCs compared to non-classical immune regulators like epithelial and stromal cells.

While our results suggest novel considerations in the development of prophylactics and therapeutics for RSV, perhaps involving IDO antagonism, how these findings will translate to human infection is unclear, in part due to the results gathered from IDO-knockout mouse experiments. This work found no difference in the number of RSV PFU in the lungs of IDO-knockout versus wild type C57Bl/6 mice. One possible reason could be the reported disparity between mice and men in the immune-regulatory role of IDO. For example, in humans IDO has been shown to be induced in listeriosis and tuberculosis, however murine models of each of these diseases suggest that iNOS and not IDO is induced in mice [86]. In line with this, our results here as well as other reports [87, 88] suggest that activated murine MSCs utilize iNOS in their immune regulatory function, while human MSCs exert their effects via IDO under the same stimuli. This difference in IDO and iNOS expression by stromal cells may play a role in the dissimilarities seen in RSV replication and response between mice and humans and also may account for the inability of the murine model of RSV infection to fully recapitulate the human disease [89–91]. Recently a group reported the use of a “humanized” mouse MSC model in which MSCs produced IDO under the control of the iNOS promoter [92]. Given these results showing the importance of MSCs in RSV pathogenesis and anti-RSV immunity a similar strategy may be useful to further elucidate the importance of IDO produced by RSV-infected MSCs.

Overall, these results provide the first report that RSV infection of bronchial epithelial cells and MSCs results in expression of the immune regulatory molecule IDO, which may be involved in disrupting the development of protective immunity against subsequent infections with the virus. Inhibition of virus-induced IDO production or activity may be a viable target for developing more effective therapies to respiratory viral pathogens. These results have implications beyond RSV-induced lung diseases, since the use of MSCs is being investigated in several areas of medicine including reducing rejection rates in solid organ transplantation, as cell-based therapy for autoimmune disease, as well as use in regenerative medicine [28, 33, 37, 69, 93]. A database search at ClinicalTrials.gov for the terms “Mesenchymal Stem Cells” returned 304 open studies (searched 10/14/15) demonstrating the high level of interest in the use of these cells in a clinical setting. This underscores the importance of understanding the consequences of RSV infection in this cell population in addition to understanding the mechanisms behind viral enhancement of their immune regulatory function, which may lead to procedures that optimize this feature for regulatory or regenerative applications.
